# Perioperative external ventricular drainage vs. no-EVD strategy in pediatric posterior fossa tumors—pilot study results

**DOI:** 10.1007/s00381-022-05819-7

**Published:** 2023-01-13

**Authors:** Matthias Krause, Jürgen Meixensberger, Hagen Graf von Einsiedel, Daniel Gräfe, Ulf Nestler

**Affiliations:** 1 Department of Neurosurgery, Pediatric Neurosurgery, University Hospital Leipzig, University Leipzig, Liebigstrasse 20, Leipzig, 04103 Germany; 2 Department of Pediatric Oncology and Hematology, University Leipzig, Leipzig, Germany; 3 Department of Pediatric Radiology, University Leipzig, Leipzig, Germany

**Keywords:** Hydrocephalus, Pediatric posterior fossa tumor, External ventricular drainage, CSF diversion

## Abstract

**Introduction:**

Pediatric brain tumors of the posterior fossa often present with occlusive hydrocephalus. Endoscopic third ventriculostomy (ETV) or ventriculoperitoneal shunting (VPS) has been established for definite hydrocephalus treatment. The aim of the study was to analyze the impact and safety of perioperative temporary external ventricular CSF drainage (EVD) placement on postoperative hydrocephalus outcome compared to a no-EVD strategy.

**Patients and methods:**

In a prospective database, 36 posterior fossa tumor patients of 2–18 years were included with a follow-up of 1 year. Fifty-eight percent presented with preoperative hydrocephalus. Patients were assigned to non-hydrocephalus group: group I (*n* = 15) and to preoperative hydrocephalus, group IIa with EVD placement (*n* = 9), and group IIb without EVD (*n* = 12).

**Results:**

Median age of patients was 8.1 years (range 3.17 to 16.58 years). One-third of 21 hydrocephalus patients required ETV or VPS (*n* = 7). Occurrence of de novo hydrocephalus in group I after surgery was not observed in our cohort. Age and histology were no confounding factor for EVD placement between group IIa and IIb (*p* = 0.34). The use of EVD did not result in better control of hydrocephalus compared to no-EVD patients considering pre- and postoperative MRI ventricular indices (*p* = 0.4). Perioperative placement of an EVD resulted in a threefold risk for subsequent VPS or ETV (group IIa 55.5% vs group IIb 16.6%): relative risk for EVD patients compared to no-EVD patients with hydrocephalus was 3.3 (CI = 1.06–13.43, *p* = 0.09).

**Conclusion:**

Perioperative EVD placement appears to harbor a threefold relative risk of requiring subsequent permanent CSF diversion in children above 2 years. EVD was not more effective to control ventricular enlargement compared to tumor removal alone. The no-EVD strategy was safe and did not result in postoperative complications. Thus, to evaluate potential adverse effects on hydrocephalus outcome by EVD placement, a prospective study is warranted to falsify the results.

## Introduction

Pediatric brain tumors occur within the posterior fossa in a relatively high percentage of more than one-half. According to our own published data, 26% of all pediatric patients diagnosed with CNS tumor suffer from symptomatic hydrocephalus (HC) as presenting symptom (37 of 142 children) [1]. Management of cerebrospinal fluid (CSF) circulation disorders ranges from preoperative endoscopic third ventriculocisternostomy (ETV), implantation of an external ventricular drainage (EVD) pre- or intraoperatively, and delayed postoperative ETV and ventriculoperitoneal shunting (VPS) [2]. Incidence of preoperative and postoperative hydrocephalus is rarely reported. Literature rates for VPS implantation for persistent hydrocephalus range from 9 to 33.3% after posterior fossa surgery [2–5]. Some authors found that age at time of surgery, preoperative ventricular size measured as frontal and occipital horn ratio (FOHR), and implantation of an EVD are associated with the need of permanent CSF diversion [6–8]. Some authors advocate endoscopic third ventriculostomy prior resection [9]. For prediction of hydrocephalus outcome after posterior fossa tumor surgery, the modified Canadian Preoperative Prediction Rule for Hydrocephalus (mCPPRH) was proposed [10, 11].

There is still an ongoing debate about the best treatment protocol: the use of preoperative EVD, treatment of hydrocephalus prior to tumor surgery with primary ETV, or a strategy of upfront tumor removal without any perioperative CSF diversion.

The aim of the study was to analyze whether the use of EVD placement is superior to a no-EVD strategy in terms of hydrocephalus management and perioperative safety.

## Patients and methods

All pediatric patients primarily diagnosed and treated for posterior fossa tumors at the University Hospital Leipzig between 2013 and 2019 were enrolled in a retrospective database. Inclusion criteria were pediatric patients with primary tumors without previous neurosurgical procedures with an age above 2 and below 18 years at time of presentation. Patients below the age of 2 were excluded because of the reported higher risk of permanent hydrocephalus after tumor surgery [4, 7, 8, 11].

The minimum for postoperative follow-up was set to 6 months after first surgery. Metastasis, biopsies, and previous posterior fossa surgeries in other institutions were excluded. Additionally, the mCPPRH score was calculated retrospectively.

In order to control secondary factors for hydrocephalus, patients were ruled out with one of the following comorbidities: postoperative complications, e.g., postoperative hemorrhage, malignant edema, surgical site or cerebrospinal fluid (CSF) infections, and primary leptomeningeal dissemination as cause of hydrocephalus. Patients that succumbed to the disease before reaching the end point were excluded from analysis.

Indication for EVD placement was made by an independent senior neurosurgeon (JM) without randomization depending on clinical and radiological status of the patient. EVD placement was performed via a right frontal burr hole. All patients underwent posterior fossa craniotomy and tumor removal surgery within 48 h after the diagnosis was made. All but one patient was operated by the same two neurosurgeons (MK and UN).

Besides MRI criteria, indications for permanent CSF diversion were clinical symptoms of persistent elevated intracranial pressure, occurrence of an enlarging pseudomeningocele or a therapy-resistant CSF leak. Patients encountering CSF infections were excluded from analysis. CSF infection was defined by routine clinical CSF data (lactate, cell count, glucose, and protein concentration) and/or positive bacterial culture or microscopy from CSF.

Radiologic parameters of ventricular configuration were evaluated in T2 axial MRI scans that had been obtained preoperatively and postoperatively for follow-up at 24–72 h after surgery. Third ventricular width was measured in mm. Changes in ventricular size were calculated as percentage from the baseline. Preoperative hydrocephalus was defined as third ventricular enlargement > 5 mm and/or periventricular edema of the lateral ventricles. Third ventricular width has been preferred because it is a simple, angulation insensitive, relatively robust, and age-independent value with a minimal error rate when measured near the thalamic adhesion. Additionally, frontal and occipital horn ratio (FOHR) was calculated from the same MRI scans [6].

Patients were assigned to a non-hydrocephalus group I and hydrocephalus group II according to the preoperative MRIs and the presence of clinical signs of increased intracranial pressure. Group II patients were separated into group IIa with pre- or intraoperative implantation of an external ventricular drainage (EVD) and group IIb without EVD placement. The need for VPS implantation or ETV was assessed for all three groups and the entire cohort within the follow-up period.

Radiographic and clinical data were collected in a retrospective database including results of routine CSF specimen examinations. All patients or caregivers gave their informed consent for the compilation of clinical, CSF, and radiological data. A local ethics committee approval has been granted for the database and study (Ethikkommission Universität Leipzig Az 330–13-18,112,013).

Statistical analysis for the groups was performed using Welch’s *t*-test and ANOVA analysis. Odds ratio for postoperative CSF diversions surgery was calculated using SPSS Version 24 (IBM Corp. Released 2016. IBM SPSS Statistics for Windows, Version 24.0. Armonk, NY: IBM Corp.). The significance level was set to 0.05.

## Results

A total of 39 patients have been eligible and enrolled in the study. However, two patients did not reach the primary end point because one succumbed due to rapid tumor progress and one presented with primary leptomeningeal spread. One patient died due to a malignant postoperative posterior fossa edema. The remaining 36 patients were enrolled into the analysis.

An overview of the patients group’s demographic, histological, and radiographic data is shown in Table 1. The demographic data disclosed a significant younger age in patients with preoperative hydrocephalus (median age 6.55 vs 9.22 years, *p* = 0.02). There was no age difference between patients that did not require VPS or ETV and those who did: mean age 7.09 years vs. 5.47 years (*p* = 0.13).

**Table 1:** Overview of demographic data, hydrocephalus incidence and CSF diversion surgeries. Group I: no preoperative hydrocephalus. Group II: all patients presenting with preoperative hydrocephalus. Group IIa: patients that underwent pre- or intraoperative EVD. Group IIb: patients without perioperative EVD. *p < 0.05

Group	*n*	Mean age (range)	Male:female	Histology	preOP HC	postOP HC	Shunt/ETV (%)	Mean 3rd ventricle diameter (range)	Mean FOHR (range)	Mean mCPPRH (range)
All	36	7.66 (2.34–16.58)	20/16	-	21	7	5/2 (19.4%)	8.4 (2–20)	0.38 (0.29–0.48)	3 (0–5)
I	15	9.22 (2.34–16.58)	8/7	4 MB, 6 PA, 2 E, 2 diffA, 1 VS	0	0	0	4.1* (2–8)	0.33* (0.29–0.35)	1 (0–2)
II	21	6.55 (2.69–13.78)	13/8	8 MB, 10 PA, 1 E, 1 diffA, 1 GBM	21	7	5/2 (33.3%)	11.5* (5–20)	0.41* (0.34–0.48)	4 (2–5)
IIa	9	6.22 (3.17–13.78)	5/4	4 MB, 4 PA, 1 GBM	9	5	4/1 (55.5%)	11.8 (5–20)	0.41 (0.35–0.47)	4 (2–5)
IIb	12	6.80 (2.69–11.71)	8/4	4 MB, 6 PA, 1 E, 1 diffA	12	2	1/1 (16.6%)	11.1 (7–18)	0.42 (0.34–0.48)	3 (2–4)

*preOP HC* preoperative hydrocephalus, *postOP HC* postoperative hydrocephalus, *FOHR* frontal and occipital horn ratio, *ETV* endoscopic third ventriculostomy, *mCPPRH* modified Canadian Preoperative Prediction Rule for Hydrocephalus, *MB* medulloblastoma, *PA* pilocytic astrocytoma, *E* ependymoma, *diffA* diffuse astrocytoma, *GBM* glioblastoma, *VS* vestibular schwannoma

Fifty-eight percent of all posterior fossa tumor patients (*n* = 21) presented with hydrocephalus. One-third of group II patients (19% of all patients) required a permanent CSF diversion (either VPS or ETV) during follow-up of 1 year. Newly acquired postoperative hydrocephalus did not occur in any patient of the non-hydrocephalic group I. Five VPS were implanted and two ETVs were performed between 7 days and 8 months after primary surgery.

Indications for permanent CSF diversion were signs of raised intracranial pressure (*n* = 4), CSF wound leakage (*n* = 2), and a tense pseudomeningocele (*n* = 1). One valve upgrade and one wound revision were necessary after VPS. No ETV reclosure occurred.

There was also no age difference between patients that underwent EVD placement compared to the patients without EVD (median age 6.22 vs. 6.8 years, *p* = 0.34). Histological diagnosis of the tumors was reviewed and did not disclose any preponderance of a specific malignant or benign entity in one of the subgroups.

There was no difference in preoperative ventricular width measured as 3rd ventricular diameter and FOHR between the groups IIa and IIb (*p* = 0.724 resp. *p* = 0.455). By definition, there was a highly significant difference in preoperative third ventricular diameter and FOHR between group I and II (*p* < 0.0001). The postoperative change of ventricular size within 24–72 h after tumor surgery did not differ between groups I and II (*p* = 0.129). The use of EVD (group IIa) did not result in better control of ventricular enlargement in comparison to tumor surgery alone (group IIb) (*p* = 0.4). EVD implantation did not result in smaller ventricles in postoperative MRI than removal of the tumor alone. No EVD infection occurred.

An intraoperative EVD was implanted at time of surgery in six patients and 24–48 h prior to tumor surgery in one patient. No patient of the two groups, additional EVD placement was necessary after tumor surgery. Pre- or intraoperative placement of an EVD resulted in a more than sixfold risk for requiring VPS or ETV (2 vs. 5 in groups IIa and IIb): In group IIa, 55.5% required CSF diversion compared to 16.6% in group IIb. Thus, the relative risk for persistence of hydrocephalus after tumor surgery between group IIa and IIb was 3.33 (CI = 0.82–13.4, *p* = 0.09, number needed to harm 2.5).

## Discussion

The limitations of the study are the retrospective and non-randomized design and the small number of children in the individual subgroups of the mono-center analysis.

The present study analyzed the incidence of persistent or newly acquired hydrocephalus requiring CSF diversion after posterior fossa tumor surgery. The incidence of newly acquired postoperative hydrocephalus seemed to be lower than 6% since none occurred in the 15 patients of group I. The overall CSF diversion rate is in concordance with the literature [2–4, 7].

The obvious risk factor for permanent hydrocephalus is EVD placement. However, this does not present a causal factor but a statistical association: patients that present in an endangered neurological state that necessitates EVD placement have progressed beyond the point of no return in CSF circulation and intracranial pressure decompensation already at time of presentation.

Importantly, EVD placement did not prove to be superior for controlling ventricular enlargement compared to tumor removal alone (*p* = 0.724). Secondary EVD after tumor surgery was not necessary in any patient even when preoperative hydrocephalus was present.

The higher rate of CSF diversions necessary after EVD placement may also partially be explained by other confounding factors than the use of EVD. A prolonged loss of supratentorial CSF pressure and reduced transaqueductal flow may pathophysiologically promote permanent obstruction of IVth ventricular outlets by postoperative blood and debris.

Young age has been associated with a higher risk for permanent hydrocephalus by various authors and the mCPPRH [3, 4, 8–11]. However, in concordance to the literature, young children below the age of 2 years have been excluded to rule out age as confounding factor [7, 11]. The assumption that EVD might promote persistence of CSF disturbance is underlined by the fact that the preoperative ventricular size was similar in groups IIa and IIb (*p* = 0.4), ruling out a bias of EVD placement in more severe hydrocephalic children.

Our findings are in concordance with results published by Helmbold et al. and Gopalakrishnan et al. that found perioperative EVD placement to be associated with ventriculoperitoneal shunting [4, 7].

A prophylactic ETV as advocated by some authors still appears to be controversial in light of a CSF diversion rate of 33% in all patients with preoperative hydrocephalus [9]. Interestingly, Frisoli et al. reported a similar rate of 16% for VPS placement after preoperative ETV. This is a similar incidence of CSF diversion as in our cohort without EVD (group IIb) [9]. It raises the additional question whether or not preoperative ETV lowers the need for postoperative CSF diversion.

Finally, a strategy of upfront tumor removal surgery without EVD seems justified in stable patients without clinical warning signs. This did not appear to harbor an increased risk for perioperative decompensation of hydrocephalus.

## Conclusion

Perioperative EVD placement was not superior to control ventricular enlargement compared to tumor removal alone. The no-EVD strategy was safe and did not cause any perioperative complications. However, EVD placement in our cohort was associated with a more than threefold higher relative risk for persistent hydrocephalus in posterior fossa tumor surgery.

We suggest the initiation of a larger prospective and randomized trial to falsify our findings. In light of the safety of a no-EVD strategy, randomization appears to be justified in clinically stable patients.
